# Supplementary material for: Efficient spin excitation via ultrafast damping-like torques in antiferromagnets
Source: Nat Commun. 2020 Dec 1;11:6142. doi: 10.1038/s41467-020-19749-y (PMC7708471; doi:10.1038/s41467-020-19749-y)
Supplement: Supplementary file 1 — Supplementary Information [file 41467_2020_19749_MOESM1_ESM.pdf]

Supplementary Information

Efficient spin excitation via ultrafast damping-like torques in antiferromagnets

Ch. Tzschaschel *et al.*

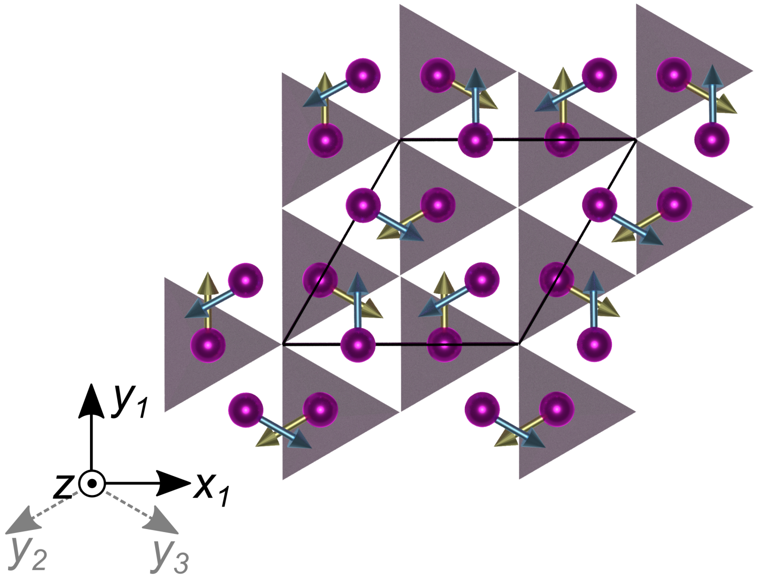

Supplementary Fig. 1. **Spin arrangement in HoMnO**$_3$ **for** $T_{\mathrm{SR}} < T < T_{\mathrm{N}}$**.** Mn^{3+} ions (violet) in grey and white areas are located in the planes at $z = 0$ and $z = c/2$, respectively. At the SRT in HoMnO$_3$, all spins in the unit cell undergo a coherent $90°$ rotation around the z axis. In comparison to Fig. 1a in the main text, the sublattice magnetisation $\mathbf{M}_i$ now points along the local y axis y_i.

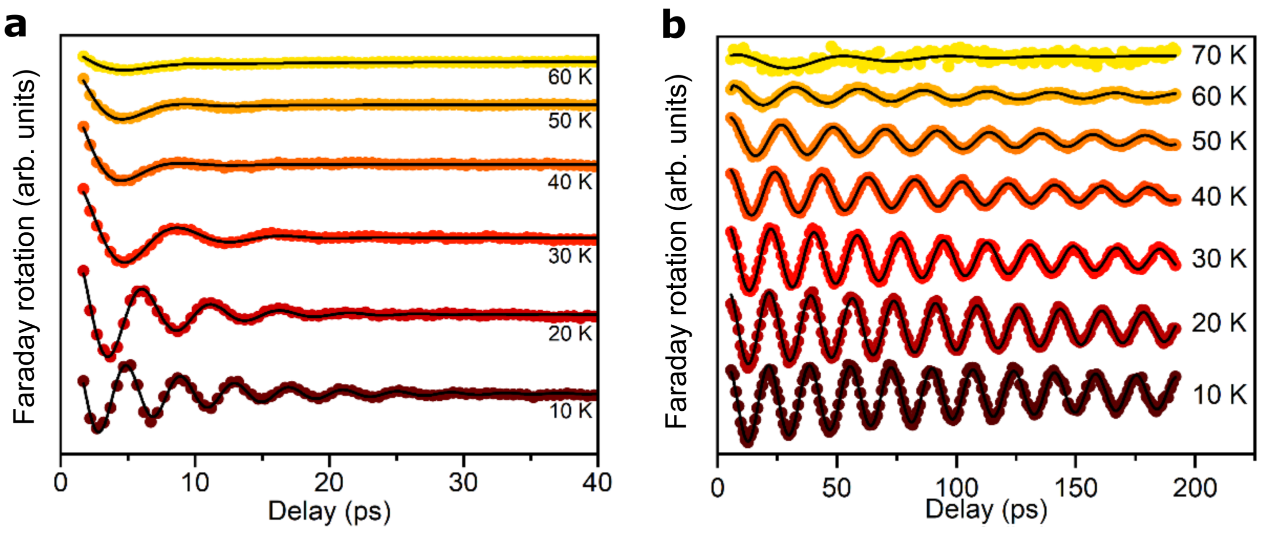

Supplementary Fig. 2. **Exemplary time-domain data** for **a** $HoMnO_3$ and **b** $YMnO_3$. The temperature-dependent softening of the spin precession frequency is clearly visible. Curves are vertically offset for clarity.

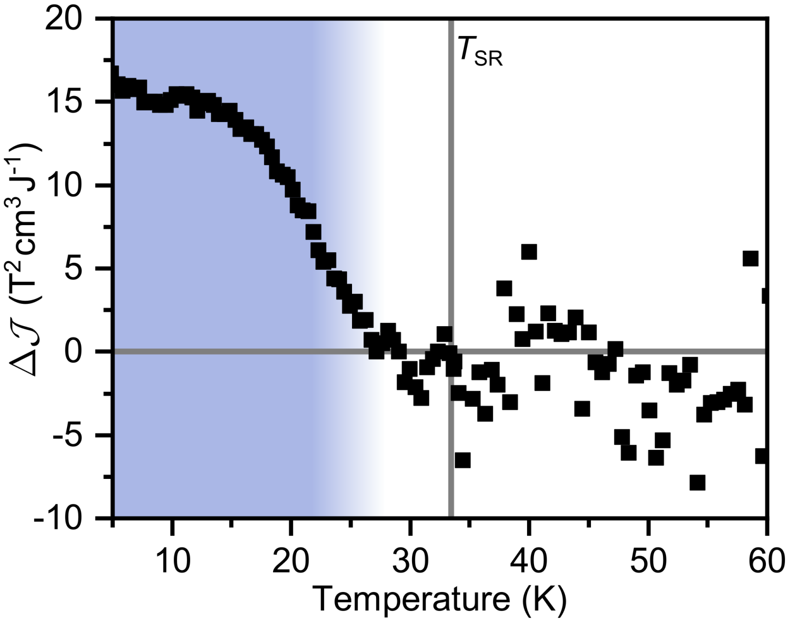

Supplementary Fig. 3. **Temperature-dependent change of $\mathcal{J}$ in $HoMnO_3$ below 25 K** The model presented in the main text for the description of spin dynamics in $HoMnO_3$ captures the temperature dependence of the Z-mode frequency, relaxation time, and initial phase well for temperatures above 25 K. Below 25 K, however, clear deviations are visible. The increased magnon frequency $\omega_0 = 2\gamma M_0\sqrt{\mathcal{J}\mathcal{D}}$ relative to the model predictions, and the decreased relaxation time $\tau \propto \sqrt{\mathcal{D}\mathcal{J}^{-1}}$ relative to the exponential temperature dependence (Figs. 3a and 3b in the main text) are both indicative of an increase in $\mathcal{J}$ rather than a change in $\mathcal{D}$ as the microscopic origin ($\mathcal{D}$ and $\mathcal{J}$ as defined in the main text or introduced in Supplementary Note 1). Assuming that $\mathcal{D}$ remains unchanged from the model presented in the main text, the change $\Delta\mathcal{J}$ can be extracted from ω_0. We find an increase in $\Delta\mathcal{J}$ by approximately 15 T^2 cm^3 J^{-1} towards lower temperatures. Incidentally, an incipient ordering of the Ho(4b) moments along the z axis was observed by neutron scattering [1]. It is rational to assume that the Ho ordering affects either the exchange interaction λ between the Mn moments or their magnetocrystalline anisotropy D_z both of which enter $\mathcal{J}$, but not $\mathcal{D}$. We therefore attribute the observed changes in ω_0 and τ to the incipient Ho(4b) ordering.

SUPPLEMENTARY NOTE 1: SPIN EXCITATIONS IN H-*R*MNO$_3$

As the spins in hexagonal $YMnO_3$ and $HoMnO_3$ are ordered within the xy plane, all antiferromagnetic domains respond identically to the effective magnetic field pulse of the IFE $\mathbf{H}_{\mathsf{IFE}} \parallel \hat{\mathbf{z}}$. We therefore assume, without loss of generality, an antiferromagnetic single-domain state and describe the magnetic order on the level of the unit cell with the free energy density of Equation (1) in the main text. As mentioned there, we neglect the weak inter-plane exchange interaction, which allows us to consider only the three spins in one lattice plane out of the six spins per unit cell, i.e. $i, j = \{1, 2, 3\}$. By defining the net magnetisation $\mathbf{M} = \sum_i \mathbf{M}_i$, we can rewrite the exchange term of the free energy density $\mathcal{F}$ as,

$$\lambda \sum_{\langle i,j \rangle} \mathbf{M}_i \cdot \mathbf{M}_j = \frac{\lambda}{2} \sum_i \mathbf{M}_i \cdot (\mathbf{M} - \mathbf{M}_i) \,. \tag{1}$$

Limiting our considerations to the Z mode, we set $\mathbf{M} = M_z \hat{\mathbf{z}}$. Furthermore, we use the fact that the Z-mode precession preserves the threefold rotational symmetry of the unit cell [2, 3]. Therefore, the three spins in one unit cell have the same z component and $M_z = 3M_{i,z}$. Equation (1) thus yields

$$\lambda \sum_{\langle i,j \rangle} \mathbf{M}_i \cdot \mathbf{M}_j = \frac{3}{2} \lambda \sum_i M_{i,z}^2 - \frac{3}{2} \lambda M_0^2. \tag{2}$$

Hence, omitting the constant contribution $\propto M_0^2 = |\mathbf{M}_i|^2$, the free energy density given in Equation (1) in the main text can be rewritten as $\mathcal{F} = \sum_i \mathcal{F}_i$ with

$$\mathcal{F}_i = \left(\frac{3}{2} \lambda + D_z \right) M_{i,z}^2 + D_y M_{i,y}^2 + \Delta M_{i,x}^2 M_{i,y}^2. \tag{3}$$

We can thus decouple the individual magnetic sublattices and describe the dynamics of the whole magnetic system by considering only one sublattice $\mathbf{M}_i$ with saturation magnetisation M_0. We will describe its dynamics by analytically solving the Landau-Lifshitz-Gilbert (LLG) equation [4]:

$$\frac{d\mathbf{M}_i}{dt} = -\gamma \mu_0 \mathbf{M}_i \times \mathbf{H}_i - \gamma \mu_0 \frac{\alpha}{M_0} \mathbf{M}_i \times (\mathbf{M}_i \times \mathbf{H}_i) \,, \tag{4}$$

where $\mu_0 \mathbf{H}_i = -\partial_{\mathbf{M}_i} \mathcal{F} = -\partial_{\mathbf{M}_i} \mathcal{F}_i$ is the effective magnetic field acting on the i-th sublattice. $\gamma = g\mu_{\mathrm{B}} \hbar^{-1} \approx 2\pi \cdot 28 \cdot 10^9 \, \mathrm{rad\,s^{-1}\,T^{-1}}$ denotes the gyromagnetic ratio. The dimensionless quantity α is the Gilbert damping parameter.

We will now linearise the LLG-equation and solve it with the initial conditions from Equation (2) in the main text. In agreement with the $\mathsf{P6_3'cm'}$ ground state symmetry, we set $m_x \approx m_0$ and $m_y, m_z \ll m_x$. Combining the effective magnetic field with the LLG-equation in Equation (4) yields, in the leading order,

$$\frac{d\mathbf{M}_i}{dt} = -2\gamma M_0 \begin{pmatrix} 0 \\ \mathcal{J} M_{i,z} + \alpha \mathcal{D} M_{i,y} \\ -\mathcal{D} M_{i,y} + \alpha \mathcal{J} M_{i,z} \end{pmatrix} \tag{5}$$

with the effective out-of-plane anisotropy $\mathcal{J} = \frac{3}{2}\lambda + D_z + |D_y|$ and in-plane anisotropy $\mathcal{D} = \Delta M_0^2 + |D_y|$. The solution for the z component is a damped sinusoidal oscillation with frequency ω, relaxation time τ, and initial phase ϕ_0, as given in the main text.

SUPPLEMENTARY REFERENCES

[1] Fabrèges, X. et al., Interplay between spin dynamics and crystal field in multiferroic compound $HoMnO_3$, Phys. Rev. B **100**, 094437 (2019).

[2] Satoh, T., Iida, R., Higuchi, T., Fiebig, M. & Shimura, T., Writing and reading of an arbitrary optical polarization state in an antiferromagnet, Nat. Photon. **9**, 25–29 (2015).

[3] Tzschaschel, Ch., Satoh, T. & Fiebig, M., Tracking the ultrafast motion of an antiferromagnetic order parameter, Nat. Commun. **10**, 3995 (2019).

[4] Landau, L. D., Pitaevskii, L. P. & Lifshitz, E. M., *Electrodynamics of Continuous Media, Second Edition: Volume 8 (Course of Theoretical Physics)* (Butterworth-Heinemann, London, 1984).
