# Supplementary material for: Efficient spin excitation via ultrafast damping-like torques in antiferromagnets
Source: Nat Commun. 2020 Dec 1;11:6142. doi: 10.1038/s41467-020-19749-y (PMC7708471; doi:10.1038/s41467-020-19749-y)
Supplement: Supplementary file 3 — Description of Additional Supplementary Files [file 41467_2020_19749_MOESM3_ESM.pdf]

Title: Supplementary Movie 1
Legend: Impulsive spin excitation considering only the field-like torque. The effective magnetic field $\mathbf{H}_{IFE}$ of the IFE induces a spin canting perpendicular to **H**. A net magnetization only occurs during the spin precession following the excitation.

Title: Supplementary Movie 2
Legend: Impulsive spin excitation via the combined effect of fieldlike and damping-like torques. The damping-like torque induces a net magnetization during the excitation: A highly efficient to handle to excite spin dynamics in antiferromagnets.
